# Intermatrix synthesis: easy technique permitting preparation of polymer-stabilized nanoparticles with desired composition and structure

**DOI:** 10.1186/1556-276X-6-343

**Published:** 2011-04-15

**Authors:** Patricia Ruiz, Jorge Macanás, María Muñoz, Dmitri N Muraviev

**Affiliations:** 1 Analytical Chemistry Division, Department of Chemistry, Universitat Autònoma de Barcelona, 08193 Bellaterra, Barcelona, Spain; 2 Chemical Engineering Department, UPC, 08222 Terrassa, Barcelona, Spain

## Abstract

The synthesis of polymer-stabilized nanoparticles (PSNPs) can be successfully carried out using intermatrix synthesis (IMS) technique, which consists in sequential loading of the functional groups of a polymer with the desired metal ions followed by nanoparticles (NPs) formation stage. After each metal-loading-NPs-formation cycle, the functional groups of the polymer appear to be regenerated. This allows for repeating the cycles to increase the NPs content or to obtain NPs with different structures and compositions (e.g. core-shell or core-sandwich). This article reports the results on the further development of the IMS technique. The formation of NPs has been shown to proceed by not only the metal reduction reaction (e.g. Cu^0^-NPs) but also by the precipitation reaction resulting in the IMS of PSNPs of metal salts (e.g. CuS-NPs).

## Introduction

The development of preparative methods for the synthesis of inorganic nanoparticles (INPs) with desired composition, structure and properties remains to be one of the hottest topics in the Nanoscience and Nanotechnology fields. Due to their nanometric dimension, both the physical and the chemical properties of INPs substantially differ from those of the respective bulk materials, what can be successfully used to improve the desired characteristics of INP-containing materials [1,2]. Stabilization of INPs in various polymeric matrices allows for preventing INPs aggregation and also for controlling their size and growth rate [3]. Moreover, the resulting nanocomposites combine the properties of both NPs and polymer matrix allowing for instance, the dispersion (or dissolution) of nanocomposites in organic solvents. The resulting INP solutions (or inks) can be used for the tailored modification of functional surfaces of electrochemical devices such as, for example, sensors. Sulfonated polyetherether ketone (SPEEK) has been shown to be an appropriate polymer matrix for the intermatrix synthesis (IMS) of metal NPs (MNPs) and due to its high stabilizing efficiency it also provides effective storage for a long period of time without any change in MNPs size. Highly stable (more than 1 year) SPEEK-MNP inks have been successfully used for modification of surfaces of electrochemical sensors [4-6].

The synthesis and application of various nanocomposites obtained by the incorporation of INPs inside a host polymer are intensively studied in both Polymer Science and Nanoscience and Nanotechnology fields [7,8]. Nanocomposites containing polymer-stabilized INPs (PSINPs) are examples of the nanocomposite materials of this type [4], which find numerous applications [5,9-15]. For example, CuS and PbS INPs-containing materials can be used as photovoltaic materials [16], quantum dots [17], or as active components in various electroanalytic devices [18,19].

The IMS technique [20-24] developed in our laboratory has proved to be successfully applicable for the easy preparation of catalytically and electrocatalytically active PSINPs of zero-valent metals (e.g. Cu, Pd, Ag and others) and various nanocomposite materials on their base in the form of membranes, resins or fibres. This technique is characterized by certain technical advantages (such as the simplicity and the aquatic chemistry-based procedures) compared with other INPs synthetic methods [7,8,25,26]. It also provides enhanced distribution of INPs near the surface of stabilizing polymer what is favourable for catalytic and electrocatalytic applications of polymer-INP-nanocomposites [24].

This study reports the results obtained by the further development of IMS technique to widen its application to new types of INP-containing nanocomposites such as, for example, those containing core-sandwich INPs and some others. Thus, our recent research on the electrochemical applications of Cu-NPs-containing nanocomposites revealed a high instability of these INPs towards oxidation in aqueous media (Ruiz P, Muñoz M, Macanás J, Muraviev DN: submitted). Taking into account that some copper compounds (such as, for example, CuS) also demonstrate catalytic activity [27,28], our research has been focused on IMS of low-solubility-metal-salt-NPs (i.e. metal sulphide NPs) and nanocomposites on their base. This communication reports the use of IMS of CuS and PbS INPs along with characterization of the electrochemical properties of the resulting nanocomposite materials.

## Experimental section

### Chemicals

Metal salts (NaBH_4_, Pb(NO_3_)_2_, Na_2_S·9H_2_O, CuSO_4_·5H_2_O, Pt(NH_3_)_4_](NO_3_)_2 _and Ru(NH_3_)_5_](NO_3_)_2 _all from Aldrich, Munich, Germany), acids and organic solvents (all from Panreac, S.A., Castellar del Vallès, Spain) were used as received. The polymer (polyetherethersulfone, PEEK, Goodfellow) was also used without any pre-treatment. Bidistilled water was used in all experiments.

### Methods

PEEK was sulfonated by following the procedure described elsewhere [29,30]. The casting of sulfonated PEEK (SPEEK) membranes was carried out from a 10% w/w solution of polymer in dimethylformamide (DMF) using a RK Paint Applicator (K Print Coat Instruments, Ltd. Litlington, Hertfordshire, United Kingdom). The IMS was applied to SPEEK membranes by sequential loading-reduction, loading-precipitation cycles or a combination of both. The loading of sulphonic groups was done using 0.1 M aqueous solutions for CuSO_4 _and Pb(NO_3_)_2 _for the first loading, and 0.014 and 0.0024 M solutions for Pt(NH_3_)_4_](NO_3_)_2 _and Ru(NH_3_)_5_](NO_3_)_2 _for the second one. For the reduction/precipitation step, an aqueous solution of either NaBH_4 _or Na_2_S was used. Samples of PSINPs-inks were prepared by dissolution of metal-loaded membranes in DMF (5% w/w) and dropwise deposited onto the surface of graphite-epoxy composite electrodes [31] (GECE) followed by air-drying at room temperature before sensor evaluation. The electrochemical characterization of INP-modified electrodes was carried out by a chronoamperometric technique, where a constant potential (-250 mV) in an acetic/acetate buffer media (pH 5) was applied. The calibration curves were obtained by measuring the intensity after consecutives additions of H_2_O_2 _known concentrations.

Diluted PSINPs-inks were also used for transmission electron microscopy (TEM) characterization by deposition of an ink drop onto a TEM grid followed by solvent evaporation.

### Instrumentation

The metal content inside SPEEK membranes was determined using Inductively Coupled Plasma Optical Emission Spectroscopy (ICP-OES, Iris Intrepid II XSP, Thermo Elemental). A sample (approximately 5 mg) of INP-containing nanocomposite was immersed in *aqua regia *(1 ml) for complete digestion, filtered (through a 0.22 μm Millipore filter) and adequately diluted for ICP-OES analysis. Microscopic characterization of NPs was carried out by both TEM (JEOL 2011, Jeol Ltd., Tokyo, Japan) coupled with an energy dispersive spectrometer (R-X EDS INCA) and scanning electron microscope (SEM) (Jeol JSM-6300, Jeol Ltd coupled with EDX (LINK ISIS-200, Oxford Instruments, Abingdon, Oxfordshire, United Kingdom or Hitachi S-570, Hitachi Ltd., Tokyo, Japan). To carry out the characterization of a cross section of the PbS-PSNPs-SPEEK by SEM technique, nanocomposites samples were first frozen in liquid nitrogen for improving the breaking. GECE preparation has been described previously [31]. The current intensity in amperometric detection of H_2_O_2 _was measured using a PC controlled Model 800B Electrochemical Analyzer (CH Instruments, Austin, TX, USA) supplied with an auxiliary Pt electrode 52-671 (Crison) and a Ag/AgCl reference electrode (Orion 900200).

## Results and discussion

**Figure 1:**
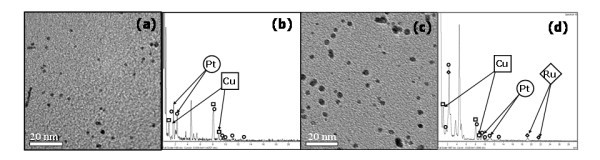
**TEM images and EDS spectra of core shell Pt@Cu- (a, b) and core sandwich Ru@Pt@Cu-PSMNPs(c, d)**.

One of the main advantages of IMS technique is the possibility of carrying out several consecutive metal-loading-reduction-cycles using the same polymer. A single metal-reduction cycle leads to the formation of monometallic NPs. However, due to the fact that the functional groups of the polymer appear to be regenerated after each cycle (converted back into the initial ionic form), undertaking consecutive cycles with another metals will result in the formation of MNPs with different structures (e.g. bi-metallic core-shell, tri-metallic core-sandwich, etc). The results presented in Figure 1 confirm this hypothesis showing TEM images and EDS spectra of bi-metallic core-shell Pt@Cu (Figure 1a, b) and tri-metallic core-sandwich Ru@Pt@Cu-PSNPs (Figure 1c, d) obtained by carrying out two and three metal-loading-reduction cycles, respectively. The results obtained agree with those reported in the literature [25] regarding simplicity and versatility of IMS technique, which provides a wide range of possibilities for obtaining INP-based nanocomposites of tuneable compositions and structures.

One additional advantage of IMS technique deals with the fact that formation of NPs proceeds mainly by the periphery of the hosting polymeric matrix due to the action of Donnan exclusion effect [24]. This distribution appears to be the most favourable in catalytic and electrocatalytic applications of INP-based nanocomposites [21,24]. Therefore, IMS technique permits to produce a high variety of catalytically active nanocomposites with high accessibility of reactants to catalytic centres.

**Figure 2:**
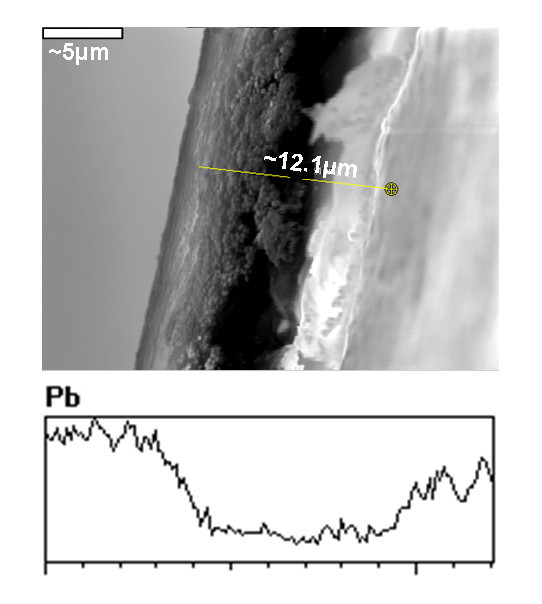
**SEM image and Pb concentration profile obtained by EDS of cross section of PbS-PSMNPs-SPEEK nanocomposite membrane**.

Furthermore, it is also noteworthy that reduction reaction (Me_1_^2+ ^+ 2BH_4_^- ^+ 6H_2_O → 7H_2_↑ + 2B(OH)_3 _+ Me_1_°) can be replaced by a precipitation reaction (Me_1_^2+ ^+ S^2- ^→ Me_1_S) if an ionic precipitating reagent bearing the charge of the same sign as that of the functional groups of the polymer (e.g. S^2-^) is used instead of a ionic reducing reagent (BH_4_^-^). As it is seen in Figure 2, the distribution of PbS-NPs obtained by IMS is similar to that for zero-valent metal NPs, i.e. PbS-NPs are mainly located near the nanocomposite sample edges. The following important conclusion follows from the results obtained: in the course of IMS of INPs when using ionic reduction or precipitation reagents, the Donnan exclusion effect appears to be the driving force responsible for the surface distribution of INPs (see EDS in Figure 2). The necessary condition in this case is the coincidence of the charge sign of ionic reagent with that of the functional groups of the hosting polymer.

**Figure 3:**
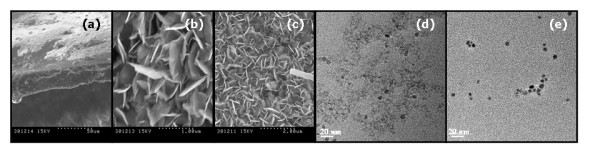
**SEM images of cross section and surface of CuS nanocomposite (a-c) and TEM images corresponding to CuS- (d) and PbS-PSNPs (e) after their dissolution in DMF**.

Figure 3a, b, c shows SEM images of a SPEEK-CuS-PSNPs nanocomposite synthesized by the precipitation version of IMS technique. As it is seen, the aggregation of CuS-NPs on the surface of supporting polymer results in the formation of a sort of nanoplates typical for CuS [32]. However, as it can be seen in Figure 3d, e, dissolution of CuS- and PbS-PSNP-containing nanocomposites in DMF leads to complete decomposition of these nanoplates into single INPs, which do not form any visible aggregates. This confirms high stabilizing efficiency of the SPEEK matrix towards INPs.

Our recent results have demonstrated that when carrying out two consecutives copper-loading-reduction cycles, the second copper-loading cycle is accompanied by the comproportionation reaction preformed after the first cycle Cu^0^-NPs and Cu^2+ ^ions from the second metal-loading solution leading to formation of Cu^+ ^ions [6]. Under optimal conditions (optimal Cu^2+ ^concentration in the second metal-loading solution), the Cu-NPs content inside the nanocomposite appears to be doubled in comparison with that obtained after one Cu-loading-reduction cycle [6].

**Figure 4:**
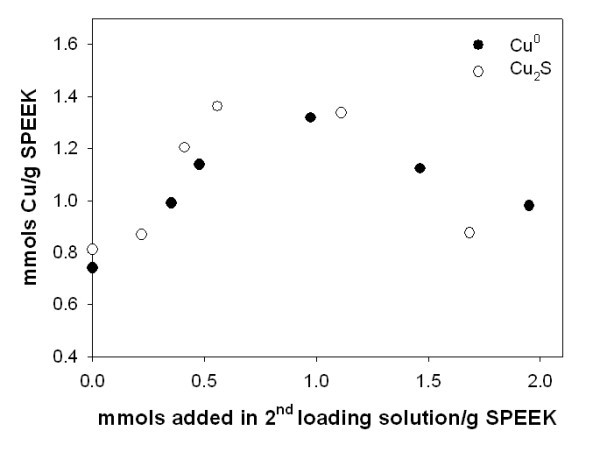
**Total Cu and Cu_2_S content in nanocomposites versus Cu mmols and in 2nd metal-loading solution**.

Figure 4 shows Cu^0^-NPs content inside the nanocomposite membrane after two metal-loading-reduction cycles and Cu_2_S-NPs content after one metal-loading reduction followed by the metal-loading-precipitation cycle. In both cases the total copper content in the membranes appears to be quite similar. At the same time, it is important to emphasize that the stability of Cu_2_S-NPs is far higher due to a far lower trend for oxidation of Cu_2_S-NPs in comparison with Cu^0^-NPs.

**Figure 5:**
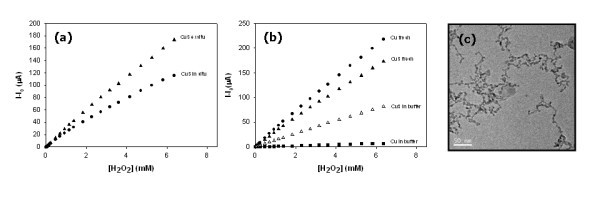
**Calibrations curves of electrochemical responses towards H_2_O_2_ with (a) CuS-PSNPs-based amperometric sensors synthesized by in situ and ex situ IMS technique and (b) with Cu and CuS-PSNPs-based amperometric sensors freshly prepared and after 3 days in buffer solution. **Experimental conditions: -250 mV; 0.1M acetic/acetate buffer, pH 5. (c) TEM images corresponding to CuS-PSNPs synthesized in situ

One of the possible applications of nanocomposite materials containing Cu_2_S-NPs is their use as catalytically active elements in electroanalytical devices such as amperometric sensors [21,23,33,34]. The sensor modification can be achieved by two different ways: (i) by depositing an ink containing INPs onto the electrode surface or (ii) by depositing the INPs-free polymeric matrix followed by the *in situ *IMS of INPs [4,21]. In the second case, the electrochemical response of the modified sensors appears to be lower than that of the sensors obtained by the *ex situ *method (see Figure 5a). TEM characterization of PSNPs prepared by *in situ *IMS shows the formation of a kind of nanowires (see Figure 5a) that could be responsible for the lower sensitivity of sensors since they are characterized by a lower surface area of INPs in comparison with well-separated spherical NPs.

In the case of sensors modified using deposition onto the electrode surface of the PMNC-ink containing Cu^0 ^or CuS (obtained after one copper-loading-precipitation cycle), reliable calibration curves were obtained for freshly prepared electrode sample in the range of 0.05-6.5 mM H_2_O_2 _as it can be seen in Figure 5b (see Cu fresh and CuS fresh curves). In order to assess the electrode stability, the INP-modified electrodes were kept in acetic/acetate buffer solution for 3 days. The results of this series of experiments are also shown in Figure 5b. As it is seen, the sensitivity of sensors modified with CuS-NPs decreases after the treatment in the buffer solution. However, the decrease of sensitivity in this case is far lower than that of sensors modified with Cu^0^-NPs after identical treatment.

## Conclusions

The main conclusion, which can be derived from the results of this study, concerns the possibility of applying the IMS technique not only for the preparation of zero-valent metal NPs but also for the synthesis of INPs of low solubility compounds (e.g. metal sulphides) using metal-loading-precipitation cycles. Another important point is the use of precipitating agents bearing the same charge as that of the functional groups of the polymer. This new version of IMS technique permits to achieve INPs distribution similar to that obtained using reduction reactions. The Donnan exclusion effect appears in both cases the main driving force responsible for this type of NPs distribution. The feasibility of preparing electroanalytical devices based on these new PMNCs has been successfully proved. The resulting amperometric sensors showed a relatively high sensitivity and a much higher stability against oxidation than those prepared using Cu -PMNCs.

## Abbreviations

DMF: dimethylformamide; GECE: graphite-epoxy composite electrodes; INPs: inorganic nanoparticles; IMS: intermatrix synthesis; MNPs: metal NPs; NPs: NanoParticles; PSINPs: polymer-stabilized INPs; PSNPs: polymer-stabilized nanoparticles; SEM: scanning electron microscope; SPEEK: sulfonated polyetherether ketone; TEM: transmission electron microscopy.

## Competing interests

The authors declare that they have no competing interests.

## Authors' contributions

PR carried out the nanocomposites synthesis and characterization. JM participated in the interpretation of the results. MM and DNM conceived of the study, and participated in its design and coordination. All authors read and approved the final manuscript.
